# The brain-skin connection: A narrative review of neuroendocrine and immune pathways

**DOI:** 10.1016/j.jdin.2025.10.008

**Published:** 2025-10-28

**Authors:** Chang Chuen Tan, Krystal Valerie Soh, Eugene Wang, Ellie Ci-En Choi

**Affiliations:** a Department of Medicine, Yong Loo Lin School of Medicine, National University of Singapore, Singapore; b Division of Dermatology, Department of Medicine, National University Hospital, Singapore

**Keywords:** acne, atopic dermatitis, bidirectional communication, brain-skin axis, cytokines, hypothalamic-pituitary-adrenal axis, immune pathways, inflammation, mental health, neuroendocrine, neuroimmune, psoriasis, psychodermatology, skin barrier, stress, Substance P

## Abstract

**Background:**

Stress is a well-established trigger for many skin diseases; yet its biological mechanisms are poorly understood.

**Objective:**

To synthesize current evidence on brain-skin pathways, emphasizing key mediators and feedback loops that explain how stress exacerbates skin disease and vice versa.

**Methods:**

A comprehensive search through August 25, 2025, using terms related to brain-skin communication, psychodermatology, and stress-related pathophysiology included both in-vivo and in-vitro animal and human studies.

**Results:**

159 articles were included and synthesized. Key findings highlight a bidirectional brain-skin axis involving the brain and pituitary, adrenal glands, peripheral nerves and skin. Stress triggers the brain and pituitary to release corticotropin-releasing hormone and adrenocorticotropic hormone; adrenal glands to secrete cortisol, catecholamines and androgens; and peripheral nerves to release neuropeptides such as Substance P and calcitonin gene-related peptide. These are implicated in skin inflammation while skin-derived mediators (cytokines, chemokines, neurotrophins) may disrupt and cross the blood-brain barrier, amplifying neuroinflammation, and psychiatric symptoms. Together, these form a self-sustaining feedback perpetuating both dermatologic and psychological disease.

**Limitations:**

Much of the mechanistic understanding is derived from animal models; high-quality human data remain limited.

**Conclusion:**

Understanding the brain-skin axis identifies therapeutic targets and guides future research on stress-related skin disease.

**Capsule Summary**

• The brain-skin axis comprises the brain, pituitary, adrenals, peripheral nerves, and skin, interconnected by neurologic, immunologic, and hormonal pathways.
• By highlighting reciprocal signaling between skin and brain, this review underscores the need for dermatologists to integrate stress management and psychosocial assessment into routine care for complex psychodermatologic conditions.

## Introduction

Stress is commonly reported as a trigger for skin diseases like atopic dermatitis, psoriasis, and acne.^1^^,^^2^ Conversely, skin diseases frequently contribute to psychological comorbidities^3^ such as anxiety and depression. Despite this naturalistic observation, few appreciate the pathophysiologic and biologic reasons for this relationship. This review summarizes the organs and mediators of the bidirectional brain-skin connection.

## Methods

We conducted a comprehensive literature search of PubMed, Embase, and Scopus from database inception to August 25, 2025.The following search string was used with minor database-specific modifications:: ((brain AND skin) OR (mind AND skin) OR (mind AND cutaneous) OR (brain AND cutaneous) OR psychoderm∗ OR psychocutaneous OR neurocutaneous) AND (connection OR axis OR signalling OR communication) AND (pathogenesis OR mechanism OR pathophysiology OR pathway) AND derm∗. Reference lists of eligible articles and relevant reviews were also hand-searched. Both in-vitro and in vivo studies, animal and human research were considered. Inclusion criteria were original articles or reviews that described mechanistic, neuroendocrine, immune, or psychodermatologic pathways linking the brain and skin. We excluded articles not available in English and those without direct relevance to brain-skin mechanisms.

Tan, Soh, and Wang independently assessed eligibility with disagreements resolved by consensus or Choi. Data were extracted and synthesized thematically, with emphasis on higher-quality in vivo human studies where available. Recurring concepts and mechanisms were consolidated into an organ- and pathway-based framework.

## Results

From 814 articles identified, 159 were included. Findings focus on key organs (brain, pituitary, adrenal glands, peripheral nerves, and skin), mediators (biochemical, hormonal, and neurotransmitters), and pathways (hypothalamic-pituitary-adrenal [HPA] axis, sympathetic-adreno-medullary [SAM] axis). We first trace brain signals downstream to the adrenals and nerves, then examine skin responses and their feedback to the brain. The principal organs involved in the brain–skin connection and their respective inputs, outputs, and interconnections are summarized in Table I.

**Table I:** Organs involved in the brain-skin connection, with their inputs, outputs and connections

Organ	Inputs	Outputs	Connections
Brain and Pituitary	Stress (internal/external)	Hormones, neuropeptides, neuronal signals	Pituitary, adrenals, spinal cord, peripheral nerves
Adrenals	Neural input, hormones (eg ACTH)	Hormones (eg cortisol, catecholamines), immune signals	Brain, skin, nerves, immune system
Peripheral nerves	Stimuli (mechanical, chemical, thermal)	Neuropeptides (eg SP, CGRP), immune cell recruitment	Brain, skin, immune system
Skin	Hormones, immune mediators (cytokines, chemokines), neurogenic mediators (neuropeptides)	ROS, cytokines, neuropeptides, stress signals, inflammation	Brain, nerves, adrenals, immune system

### Brain and pituitary

The hypothalamus activates under stress, whether from physical stressors such as surgery or chronic disease, psychologicl stressors such as conflict, grief, and cognitive overload,^4^, ^5^, ^6^, ^7^ or environmental stresses including UV light, mechanical trauma, and allergens.^8^

In response, the hypothalamus secretes corticotropin-releasing hormone (CRH),^9^ stimulating the pituitary to produce proopiomelanocortin (POMC).^10^, ^11^, ^12^ POMC is cleaved to hormones including adrenocorticotropic hormone (ACTH) and melanocyte-stimulating hormone (MSH), mediating downstream stress effects.

While acute stress may increase oxytocin levels to buffer against stress, chronic stress is associated with reduced hypothalamic oxytocin via persistently elevated cortisol,^13^ affecting emotional wellbeing, skin inflammation, and wound healing.^14^^,^^15^

Stress often disrupts sleep, regulated by the hypothalamic suprachiasmatic nucleus.^16^ Higher nocturnal cortisol can suppress melatonin, disrupting circadian rhythm, exacerbating stress response cognitively, behaviorally and pathophysiologically through production of greater proinflammatory mediators.^17^^,^^18^

The hypothalamus interprets stress signals and coordinates the sympathetic fight-or-flight response.^19^ It activates the preganglionic neurons via the paraventricular nucleus, which then stimulates sympathetic ganglia.^20^ Postganglionic fibers release norepinephrine, triggering vasoconstriction, sweating and piloerection (Table II).^20^^,^^21^

**Table II:** Brain regions and mediators involved in the stress response and its effects on skin

Brain region/structure	Function in stress response	Key mediators released	Effect on skin
Hypothalamus (Paraventricular Nucleus)	Central integrator of stress signals (physical, psychological, environmental)	CRH, Substance P, signaling to pituitary and brainstem	Initiates HPA axis and sympathetic activation → skin inflammation, mast cell degranulation
Hypothalamus (Suprachiasmatic Nucleus, SCN)	Regulates circadian rhythm and sleep-wake cycle	Melatonin (via pineal gland, regulated by SCN), indirect effect	Poor sleep from stress → ↑ pro-inflammatory cytokines → skin aging, impaired healing
Hypothalamus (Magnocellular neurons)	Regulates emotional bonding, wound healing	Oxytocin (via posterior pituitary)	Acute stress → ↑ oxytocin (protective); chronic stress → ↓ oxytocin → delayed healing, ↑ inflammation
Anterior Pituitary	Activated by CRH → produces ACTH	ACTH → stimulates adrenal cortex	↑ Cortisol → impairs barrier function, promotes pro-inflammatory milieu
Brainstem (Rostral Ventrolateral Medulla)	Coordinates autonomic response	Sends excitatory input to spinal sympathetic neurons	Triggers sympathetic activity → skin vasoconstriction, sweat production, piloerection
Spinal Cord → Sympathetic Chain Ganglia	Preganglionic to postganglionic relay of stress signals	Norepinephrine from postganglionic neurons	Vasoconstriction, impaired healing, sweat gland activation, itch/pain amplification
Blood-Brain Barrier (BBB)	Normally limits cytokine access to CNS	Disrupted under chronic stress/inflammation	Allows skin-derived cytokines to access brain → mood disorders, perpetuates stress loop

### Adrenals

The adrenals (part of the neuroendocrine system) integrate signals^22^ from both the nervous and endocrine system to regulate stress response.

Stress perceived by the brain stimulates neurons in the sympathetic nervous system, activating postganglionic fibres^23^ in the adrenal medulla to secrete catecholamines. They bind to adrenergic receptors in end organs including brain, heart, vessels and sweat glands, causing effects^24^ such as increased heart rate, peripheral vasoconstriction and sweating. Beyond these systemic effects, catecholamines modulate immune function by influencing cytokine production, immune cell trafficking, and inflammatory responses, linking sympathetic activation to immune regulation.^25^

ACTH, produced from the anterior pituitary and to a lesser degree by epidermal keratinocytes and melanocytes^26^ act on the adrenal glands, stimulating glucocorticoid synthesis^27^ like cortisol, and adrenal androgens^28^ like dehydroepiandrosterone (DHEA) and androstenedione. Cortisol increases gluconeogenesis and enhances cardiovascular function. However, it suppresses immune activity as part of the fight-or-flight response to stress,^29^ affecting skin barrier and creates a pro-inflammatory milieu which will be elaborated later. DHEA and androstenedione, converted locally in the skin to more potent androgens like testosterone and dihydrotestosterone (DHT), influence sebaceous gland activity, collagen synthesis, and inflammatory responses.^30^

### Peripheral nerves and related neurotransmitters

The skin is innervated by sensory neurons, part of the peripheral nervous system (PNS). The PNS is instrumental in how stress impacts skin inflammation by linking sensory signals, immune responses, and autonomic regulation.^31^

Sensory neurons detect various stimuli; mechanoreceptors to physical pressure, thermoreceptors to temperature changes, nociceptors to pain and chemoreceptors to chemical signals.^32^ Stress or injury activates sensory neurons, particularly nociceptors, triggering neurogenic inflammation through release of neuropeptides including Substance P, calcitonin gene-related peptide (CGRP), neurokinin A and vasoactive intestinal peptide (VIP), causing vasodilation, mast cell degranulation, and immune cell recruitment.^33^, ^34^, ^35^

Immune cells recruited to inflammation site cause release of cytokines (eg,TNF-α, IL-1β, IL-6^36^) and nerve growth factor (NGF), amplifying neural and immune responses,^34^^,^^37^ and regulating nerve regeneration and repair following injury by influencing macrophage and neuronal activity, facilitating processes such as debris clearance, axon growth, remyelination, and skin cell proliferation.^38^, ^39^, ^40^ Pro-inflammatory cytokines also sensitize sensory neurons in the skin, increasing pain perceptions.^41^^,^^42^

### Skin

The skin functions as an active endocrine, immune, and sensory organ that responds to external stressors on the skin (eg, UV radiation), hormonal, immune and neurogenic mediators,^8^^,^^43^, ^44^, ^45^ regulating inflammation, immune function, and barrier integrity.^46^^,^^47^

#### Hormonal mediators

Hormones, including CRH, ACTH, cortisol, catecholamines, androgens are chemical messengers part of the HPA axis that circulates in the bloodstream.^48^ A peripheral HPA axis exists in parallel within the skin where these hormones are locally produced by keratinocytes, mast cells, sebocytes and melanocytes. It mirrors central stress responses but acts locally in an auto-, para- and intracrine manner to regulate inflammation.^49^

CRH links psychological stress to cutaneous inflammation^50^ by activating mast cells, triggering degranulation of pro-inflammatory mediators including histamine, TNF-α, and IL-6, increasing vascular permeability^51^ and facilitating immune cell infiltration and inflammation.^52^ Additionally, CRH stimulates sebocytes^53^ to release pro-inflammatory cytokines IL-6^54^ and IL-8,^53^ driving conditions like acne and seborrheic dermatitis. Beyond its immune and sebaceous effects, CRH upregulates matrix metalloproteinases that degrade extracellular matrix proteins, contributing to impaired wound healing,^1^ increased skin fragility,^55^ and accelerated aging^55^^,^^56^ under chronic stress conditions. Furthermore, CRH enhances neurogenic inflammation by promoting neuropeptide release (eg Substance P) from peripheral nerve fibers.^57^

CRH initiates a positive feedback loop by promoting local cortisol synthesis^58^ which binds to glucocorticoid receptors on skin cells, reinforcing CRH release and sustaining chronic inflammation.^58^ Cortisol’s downstream effects include increasing ROS, reducing epidermal lipids, hyaluronic acid, type 1 collagen and stratum corneum hydration, all of which weaken the skin barrier,^59^ increasing susceptibility to irritation, dryness, infection and impaired wound healing.^60^^,^^61^

The adrenal medulla secretes catecholamines, predominantly epinephrine and norepinephrine, with minor amounts of dopamine, acting on α- and β-adrenergic receptors in keratinocytes,^62^ immune cells,^63^ endothelial cells,^64^ and sebocytes.^26^ Their skin effects are vasoconstriction, immune modulation, and sebaceous gland regulation.^63^^,^^65^, ^66^, ^67^ Vasoconstriction reduces cutaneous blood flow, which may contribute to hypoxia, oxidative stress, and delayed wound healing.^68^^,^^69^ The stress-induced ischemia triggers pro-inflammatory cytokine release (TNF-α, IL-1β, IL-6),^70^ worsening inflammatory skin conditions like psoriasis,^71^ atopic dermatitis (AD)^72^^,^^73^ and vitiligo.^74^ In AD, despite increased catecholaminergic signals, sweat gland activity is reduced due to structural or dysfunctional changes, affecting epidermal hydration, leading to skin barrier dysfunction.^75^^,^^76^ Catecholamines also stimulate sebum production, linking stress to acne pathogenesis.^44^^,^^77^

Androgens (testosterone and DHT) bind to androgen receptors on sebocytes, keratinocytes, and fibroblasts,^30^^,^^78^^,^^79^ increasing sebum production, triggering pro-inflammatory cytokine release.^30^^,^^78^, ^79^, ^80^, ^81^, ^82^

#### Immune mediators

Immune mediators like cytokines, chemokines, and bioactive lipids further shape the skin’s stress response.

Cytokines from keratinocytes, immune cells (eg, mast cells, macrophages, and T cells), and fibroblasts orchestrate inflammation in response to stress, injury, and infection.^83^^,^^84^ Pro-inflammatory cytokines (IL-1, IL-6, TNF-α, IL-17) is implicated in leukocyte recruitment,^85^ vascular permeability,^86^ and immune activation, crucial for pathogen defense and wound healing.^87^ Conversely, IL-10, an anti-inflammatory cytokine, counterbalances excessive inflammation, maintaining homeostasis.^88^^,^^89^ Chronic stress is linked to overall immune system dysregulation. It increases blood levels of pro-inflammatory cytokines and reduces anti-inflammatory cytokines via HPA and sympathetic nervous system activation,^61^^,^^90^ shifts T-helper balance towards Th2 dominance, and impairs immune cell maturation and wound healing. This shift exacerbates sebaceous gland inflammation, worsening acne, psoriasis, and chronic skin conditions.^1^ Additionally, cytokines contribute to collagen degradation and accelerated aging, linking stress to skin atrophy and barrier dysfunction.^91^

Dysregulated cytokine signaling is a shared feature in dermatological and psychiatric conditions with elevated cytokines IL-4 and IL-13 in AD associated with depressive symptoms,^92^, ^93^, ^94^, ^95^ IL-17A has been linked to antidepressant resistance^96^ and TNF-α and IL-23 elevation with generalized anxiety disorder.^97^

Chemokines like CCL2 and CXCL8 are upregulated, guiding immune cell migration to both the skin and brain, contributing to skin inflammation and psychiatric disorders. Bioactive lipids like prostaglandins and leukotrienes worsen inflammatory skin condition and contribute to itch, vasodilation, and barrier dysfunction.

#### Neurogenic mediators

Neurogenic mediators include proteins like neuropeptides (Substance P, CGRP, VIP) that modulate the immune response and neurotrophins (NGF), which promote the growth of neurons.^46^

The most prominent neurotrophin is NGF, produced by keratinocytes, mast cells, fibroblasts and immune cells^98^ under stress. NGF enhances C-fiber and Aδ-fiber activity, intensifying itch and pain sensitivity.^99^ While NGF promotes keratinocyte proliferation and wound healing,^100^^,^^101^ chronic overexpression leads to hyperproliferation, sebaceous gland hyperactivity, impaired barrier function, worsening psoriasis^102^ and stress-induced skin aging.^103^ Stress-driven neurotrophin dysregulation reinforces neurogenic inflammation,^37^ sustaining a pro-inflammatory loop between the nervous and immune systems and influence neuropeptide release.^103^

Substance P and CGRP released from sensory nerves and skin cells is implicated in neurogenic inflammation.^104^ Substance P, a neuropeptide, enhances the body’s immediate defense to noxious stimuli and stress. It activates NK-1R on mast cells, endothelial cells, and immune cells triggering mast cell degranulation, vasodilation, and cytokine release, contributing to cutaneous inflammation.^104^, ^105^, ^106^ CGRP targets endothelial, smooth muscle and immune cells,^107^^,^^108^ promoting vasodilation, increased vascular permeability, and modulates immune signaling, reinforcing stress-induced skin responses.^109^^,^^110^ Both neuropeptides are instrumental in pain and itch perception,^111^ acting synergistically to amplify sensory discomfort in inflammatory skin conditions.^112^

#### Other mediators

Antimicrobial peptides produced in the skin contribute to cutaneous innate immunity and defense against microorganisms. They are typically protective and limit excess immune activation. Chronic stress downregulates antimicrobial peptide expression via elevated cortisol, catecholamines, and neuropeptides,^113^^,^^114^ increasing susceptibility to infections, inflammation, and impaired wound healing. Their dysregulation is implicated in chronic skin conditions including atopic dermatitis, psoriasis, and rosacea.^113^^,^^115^, ^116^, ^117^

### The bidirectional brain-skin connection

The brain-to-skin pathways have been outlined above and the focus is shifted to the skin-to-brain signaling, mediated by systemic immune responses and cutaneous sensory inputs.

The BBB exhibits selective permeability to keep peripheral inflammatory signals out of the central nervous system (CNS).^118^^,^^119^ Nevertheless, pro-inflammatory cytokines may alter the BBB^3^, ^4^, ^5^, ^6^, ^7^ impairing serotonin, dopamine, and norepinephrine pathways.^8^^,^^9^ Elevated IL-17 and IL-22 weakens the BBB by disrupting tight junctions, facilitating immune cell infiltration and sustaining neuroinflammation in chronic skin diseases like psoriasis. These cytokines are elevated in conditions like alopecia areata,^120^^,^^121^ acne,^122^ and atopic dermatitis,^91^^,^^123^, ^124^, ^125^ are associated with a higher prevalence of anxiety, depression, cognitive decline, and neurodegenerative diseases.^10^, ^11^, ^12^, ^13^ Chronic stress may disrupt the BBB, contributing to neuroinflammation and changes in neurotransmitter systems.^14^^,^^119^^,^^126^ The compromised BBB reinforces the skin-to-brain signaling loop and perpetuates stress-related dermatological and psychiatric comorbidity.^127^

Furthermore, skin conditions carry high symptom burden and morbidity from both the physical symptoms and visible skin rashes. Emotionally, individuals with skin diseases experience higher rates of depression, anxiety and reduced life satisfaction.^3^^,^^128^^,^^129^ The psychological comorbidities are associated with disease characteristics including chronicity and psychobehavioral constructs like coping style and personality.^130^^,^^131^ Socially, skin diseases can be both stigmatizing and self-stigmatizing.^132^ Individuals with visible skin conditions may internalize negative perceptions, contributing to reduced self-esteem, social anxiety and depression. Public misconceptions on conditions like hidradenitis suppurativa, psoriasis and eczema being caused by poor hygiene or are contagious can lead to external stigma and workplace and social discrimination, further impacting symptom burden and psychological comorbidities.^133^, ^134^, ^135^, ^136^, ^137^, ^138^

The psychosocial distress in turn activates the immune-endocrine pathways discussed previously. Studies show elevated cortisol and catecholamines with chronic emotional stress, anxiety, and depression, contributing to increased production of pro-inflammatory cytokines (eg, IL-6, TNF-α, and CRP) which impair immune homeostasis, creating a systemic pro-inflammatory state that both exacerbates dermatological disease activity and neuroinflammation.^139^^,^^140^

### Emerging variables

Beyond classical neuroendocrine and immune pathways, several emerging factors may shape the brain-skin connection. Genetic studies, including genome-wide association and Mendelian randomization analyses, suggest shared susceptibility loci between psychiatric disorders (eg, depression, anxiety, and schizophrenia) and immune-mediated skin diseases like psoriasis and atopic dermatitis.^141^, ^142^, ^143^ Stress can also alter eating habits and the gut-skin microbiome, reducing antimicrobial defenses and promoting pathogenic bacterial growth and biofilm formation.^1^^,^^144^, ^145^, ^146^ In parallel, psychosocial variables like coping style, resilience, and perceived stigma may modulate neuroimmune responses, further influencing disease expression.^1^^,^^147^, ^148^, ^149^ While still preliminary, these findings highlight the importance of integrating genetic, microbial, and behavioral perspectives in future brain-skin research.^43^^,^^143^^,^^150^, ^151^, ^152^

## Discussion

Taken together, the evidence supports a mind-skin connection mediated by biological, psychological, and behavioral pathways. The interplay between immune dysregulation, emotional distress, and lifestyle factors suggests management likely requires a multimodal approach targeting inflammatory (eg, immunosuppressants and biologics), neuronal (eg, anticonvulsants, κ-opioid receptor agonists, and μ-opioid receptor antagonists), and psychobehavioral (eg, cognitive behavioral therapy and habit reversal training^153^) pathways.

This review highlights a lack of robust mechanistic human studies. Much of the literature is based on animal or in-vitro models, and human studies are mainly small, cross-sectional, and observational, limiting causal inference. Furthermore, most studies focused on individual systems in isolation, such as the HPA axis, providing insufficient data on the complex, interconnected neuroimmune and neuroendocrine pathways. We did not perform a formal quality appraisal given the wide heterogeneity of methodologies included; however, it is important to note that overall evidence strength remains limited, with few randomized trials or meta-analyses currently available.

**Fig 1:**
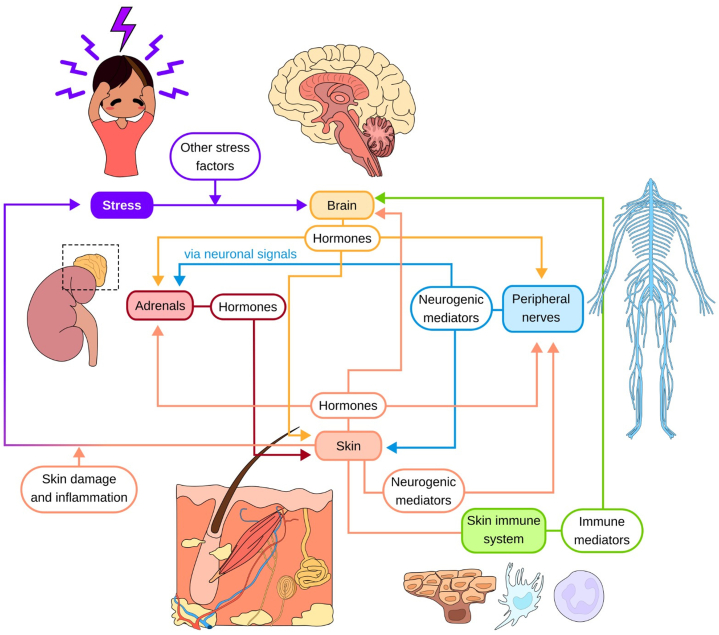
Diagram of Brain-Skin connection involving neuroendocrine and immune pathways. Colored lines represent mediator types: yellow for hormonal, blue for neurogenic, green for immune.

This review is not exhaustive; mediators of skin inflammation like arachidonic acid and nitric oxide were excluded due to insufficient evidence directly linking them to the brain-skin axis. Psychosocial variables, genetic predispositions, and lifestyle factors, though relevant, were beyond the scope of this review. Hence, our synthesis emphasizes the best-characterized neuroendocrine and immune pathways, presented through an organ-based framework tracing signals between brain and skin (Fig 1). Future studies that integrate these broader dimensions will be critical to refining our understanding of the evolving brain-skin field.

Future longitudinal studies could examine how pharmacological or psychotherapeutic interventions modify systemic and neuroimmune signatures. Observational research, though valuable, cannot distinguish causation from correlation due to shared risk factors. Encouragingly, experimental studies are increasingly feasible. The growing affordability of omics platforms and advances in stress-monitoring tools like CARES sensors and galvanic skin response/electrodermal activity devices may enable controlled experiments that directly induce and quantify stress responses in the skin.^154^, ^155^, ^156^, ^157^

## Conflicts of interest

None disclosed.
